# Cervical cancer immune infiltration microenvironment identification, construction of immune scores, assisting patient prognosis and immunotherapy

**DOI:** 10.3389/fimmu.2023.1135657

**Published:** 2023-03-10

**Authors:** Shijie Yao, Liyang Zhao, Siming Chen, Hua Wang, Yang Gao, Ning-Yi Shao, Mengyuan Dai, Hongbing Cai

**Affiliations:** ^1^ Department of Gynecological Oncology, Zhongnan Hospital of Wuhan University, Wuhan, Hubei, China; ^2^ Hubei Key Laboratory of Tumor Biological Behaviors, Wuhan, Hubei, China; ^3^ Hubei Cancer Clinical Study Center, Wuhan, Hubei, China; ^4^ Department of Biomedical Sciences, Faculty of Health Sciences, University of Macau, Taipa, Macau, Macau SAR, China; ^5^ Ministry of Education (MoE) Frontiers Science Center for Precision Oncology, University of Macau, Taipa, Macau, Macau SAR, China; ^6^ Department of Urology, Zhongnan Hospital of Wuhan University, Wuhan, China

**Keywords:** ICI score, cervical cancer, TMB, immune infiltration, immune microenvironment

## Abstract

**Background:**

The immune microenvironment is of great significance in cervical cancer. However, there is still a lack of systematic research on the immune infiltration environment of cervical cancer.

**Methods:**

We obtained cervical cancer transcriptome data and clinical information from the Cancer Genome Atlas (TCGA) and the Gene Expression Omnibus (GEO) databases, evaluated the immune microenvironment of cervical cancer, determined immune subsets, constructed an immune cell infiltration scoring system, screened key immune-related genes, and performed single-cell data analysis and cell function analysis of key genes.

**Results:**

We combined the TCGA and GEO data sets and obtained three different immune cell populations. We obtained two gene clusters, extracted 119 differential genes, and established an immune cell infiltration (ICI) scoring system. Finally, three key genes, IL1B, CST7, and ITGA5, were identified, and single-cell sequencing data were mined to distribute these key genes in different cell types. By up-regulating CST7 and down-regulating IL1B and ITGA5, cervical cancer cells’ proliferation ability and invasion ability were successfully reduced.

**Conclusion:**

We conducted a comprehensive assessment of the state of the tumor immune microenvironment in cervical cancer, constructed the ICI scoring system, and identified the ICI scoring system as a potential indicator of susceptibility to immunotherapy for cervical cancer, identifying key genes suggesting that IL1B, CST7, and ITGA5 play an essential role in cervical cancer.

## Introduction

With almost 311,000 women dying from cervical cancer in 2018 (1), it is one of the most prevalent malignancies in humans (2) and one of the leading global causes of mortality for women (3). Cervical cancer also has a substantial influence on the physical and mental health of women. It is generally recognized that the primary causes of cervical cancer are chronic HPV infection and failure of HPV clearance (4). Early injection of the HPV vaccine can effectively prevent cervical cancer (5, 6). However, it does not remove the HPV that has already been infected (7). Surgery, radiation, and chemotherapy are the most prevalent treatment options for cervical cancer, depending on the stage (8). However, most patients are already in critical condition when diagnosed (9, 10) and have missed the window for surgical intervention (11). The prognosis for people with metastatic and recurring forms of the illness remains dismal despite improvements in cervical cancer detection, treatment, diagnosis, and prevention (12). Additionally, a patient’s heterogeneity may cause certain cervical cancer patients’ resistance to immunotherapy, targeted therapy, and chemotherapy (13). Finding new therapeutic targets to improve cervical cancer patients’ prognoses is thus essential (14, 15).

There is mounting evidence that cancer and immune microenvironment modification are tightly connected (16–18). Immune cells and tumor cells interact, and an imbalance between them controls the formation of tumors. All forms of cancer are characterized by immunological escape from immune monitoring (19). One of the critical mechanisms of immunological escape is thought to be the activation of immune checkpoints. Immune checkpoint inhibitors are regarded as a successful treatment strategy, and Immunotherapy has a smaller off-target effect than chemotherapy drugs (20). Immune checkpoint inhibitors have now received FDA approval for first- and second-line treatment for various cancers (21), and favorable effectiveness has been seen in patients with metastasis and recurrence (22), such as the cytokines interferon-alpha (20), the interleukin-2 (IL-2) (23). Immunotherapy has grown in importance as a treatment for cervical cancer (24–26); precise regulation of immune targets can maximize its effectiveness (27, 28). For instance, activating immunological targets makes it easier for T lymphocytes to get activated, which is beneficial for immunotherapy, for example, cytotoxic T lymphocyte-associated protein 4 (CTLA-4) and programmed cell death 1 (PD-1) (25). A novel technique for assessing the tumor immune microenvironment was also established by Yoshihara et al. (29, 30), which is more beneficial for tumor immunotherapy and for determining which patients may benefit from immunotherapy.

The TCGA and GEO databases provided transcriptome and clinical data on patients with cervical cancer for this investigation. We measured and assessed the immune microenvironment of cervical cancer and performed a cluster analysis on the patient population using the ICI score. The intrinsic association of gene mutations was assessed using the ICI score group to forecast the possible chemical advantages of immunotherapy for cervical cancer patients.

## Methods

### Cervical cancer data collection and collation

We retrieved the Cancer Genome Atlas (TCGA) (https://portal.gdc.cancer.gov/) database’s microarray data set and associated clinical information for cervical cancer, and 306 cervical cancer samples were utilized for further investigation. The Gene Expression Omnibus (GEO) database was searched for, and the microarray data set for the GSE30759 chip was acquired; it comprises data on 48 cervical cancer patients. Only cervical cancer patients with comprehensive data, such as status and overall survival time, were gathered. Transcripts per kilobase million (TPM value) of the microarray data set (FPKM value) were translated to preserve compatibility between the two databases. The original data of the GSE30759 chip obtained from the GEO database was unified, and then the gene probes were annotated.

### Immune cell clustering of tumor immune cell microenvironment

Combined TCGA and GEO databases to assess cervical cancer’s tumor immune microenvironment level. Based on 547 gene expression values, the proportion of cervical cancer samples with mixed cell types in 22 different immune cell subpopulations was evaluated using the CIBERSORT algorithm to obtain the ICI score matrix for different immune cells. Used the R package “ConsensuClusterPlus” for analysis and iterated 1,000 times to obtain stable classification results. Analyzed the distinctive characteristics of the stromal and immune cell transcription patterns in cervical cancer and deduced tumor cellularity and tumor purity using the ESTIMATE algorithm.

### Acquisition of DEGs and construction of ICI scoring system

Using the R package “limma,” differentially expressed genes (DEGs) were found, where the absolute folding change>1 and the critical value standard P<0.05 were considered to be statistically different. The data were split into different genomic clusters according to the DEGs value using unsupervised clustering. Gene features are positively correlated with gene cluster A, whereas gene characteristics are negatively correlated with gene cluster B. The first principal component (PC1) produced by principal component analysis (PCA), after dimensionality reduction analysis using the Boruta approach, was chosen as the feature score, two PC1 values were obtained for each sample, the optimal cutoff value was taken as the dividing line and divided into high ICI score group or low ICI score group. ICI scores were generated for each instance using the gene expression rank index approach, using the formula:


$$\text{ICI score}=\sum^{}\text{PC}1\text{A}-\sum^{}\text{PC}1\text{B}$$

### Gene enrichment analysis

Gene ontology (GO) analysis was carried out on the two gene clusters to understand their biological roles better. Gene Set Enrichment Analysis (GSEA) based on two ICI score grouping, P<0.05, was determine statistically different.

### Genetic mutation data acquisition

The TCGA database gathered data on somatic mutations in cervical cancer. Data on somatic mutations were examined and categorized using the ICI score system. The “maftools” package was used to identify the 20 mutated genes in both mutation data sets.

### Drug sensitivity prediction

The Genomics of Drug Sensitivity in Cancer (GDSC) website (https://www.cancerrxgene.org/) provided information on the drug’s predicted half-maximal inhibitory concentration (IC50), and variations in drug sensitivity to Cisplatin, 5-Fluorouracil, Vinblastine, Vincristine, Paclitaxel, Docetaxel, Gemcitabine, Cyclophosphamide, Topotecan, Epirubicin, Olaparib, Dactinomycin were evaluated among ICI score groups.

### Collection of cervical cancer tissue samples

Cervical cancer tissue samples were collected from patients before receiving immunotherapy at Wuhan University Central South Hospital. Tissues were fixed with formalin and then embedded with paraffin. All samples were stored at room temperature 20°C-25°C. All specimens were diagnosed and agreed upon by at least two pathologists based on pathological features. Finally, this study included 16 cases of cervical cancer tissues that had received immunotherapy. The Zhongnan Hospital of Wuhan University ethical committee approved the research (ethics number: 2020029).

### Immunohistochemical staining

Cervical cancer tissues were fixed with formalin, followed by paraffin embedding to obtain 4um thick sections of tumor tissue. Paraffin-embedded portions were dewaxed with xylene and ethanol, hydrated and blocked paraffin sections. Sections were treated with primary antibodies, which were then incubated with them overnight in a moist box kept at 4°C. After that, PBS was used to wash the parts three times for a total of eight minutes each. The sections were then treated with secondary antibodies for 30 minutes at room temperature before being rinsed with PBS. Visualized with 3,5-diaminobenzidine (DAB) substrate kit and restrained with hematoxylin for 2 min. We divided the staining intensity of CD8A, CXCL10 and GZMB into 1, 2, 3 or 4. The stronger the staining, the higher the score. Two pathologists evaluated the expression of CD8A, CXCL10, and GZMB in the tissue microarrays in a blind manner.

### Multiplexed fluorescence immunohistochemistry

Xylene was used to dewax paraffin-embedded (FFPE) tissue sections (4 m) before they were hydrated with a series of graded ethanol solutions in deionized water. Blocking was performed by adding 3% H_2_O_2_ and left for 25min at room temperature protected from light, then rinsed three times with PBS for 5min each. 3% BSA was added and incubated for 30min. Serial staining was performed with the following antibodies, CD3, CD4, CXCL10, CD8A, and GZMB, and incubation was maintained overnight at 4°C. Next, the sections were shaken in PBST (pH 7.4) for three washes, each lasting 8 min. Anti-rabbit polymeric horseradish peroxidase-labeled secondary antibody was subsequently added and incubated for 50 min, protected from light. Tyrosine-CY3 was then introduced and continued to work for 20 minutes. Three PBST washes were then performed, each lasting for five minutes. DAPI staining solution was added to stain nuclei for 10 min. Sections were sealed with anti-fluorescence quenching sealer. Under the excitation of UV light, blue light represents DAPI-stained nuclei, and red light, green light, and pink light represent the corresponding fluorescein-labeled nuclei, respectively.

### Construction and evaluation of risk score prediction model

Univariate Cox regression analysis was used to assess each DEG’s expression levels. Multivariate Cox regression analysis was used to identify the three key genes, CST7, IL1B, and ITGA5, with the greatest predictive power. Patients were divided into high and low risk groups based on the results of the median risk assessment. Kaplan-Meier curves were used to describe overall survival rates, and the precision of the model’s predictions was evaluated using the AUC of the ROC curve.

### Single cell data analysis

Data used in this study were publically available and downloaded from the Gene Expression Omnibus and Genome Sequence Archive (GSE168652) (31), included two samples: tumor and normal), and Seurat (32) was used to perform the next analysis. Low-quality cells were filtered according to the original articles and our requirement: cells with less than 200 expressed genes or > 15% of mitochondrion-derived counts or< 0.8 of the number of genes detected per UMI were removed. All cells that passed QC were normalized to identify the top 2000 high-variable genes. Mitochondrial score and ribosome score and cell cycle were regressed in the ScaleData function to remove batch effects. Then the top 20 principal components (PCs) were selected by the RunPCA function and were set in the RunUMAP function and RunTSNE function to reduce dimensions and in cell clustering. Subsequently, the main cell clusters were identified with the FindClusters function with the method “original Louvain algorithm”. Method “MAST” in FindAllMarkers function was used to identify marker genes of different clusters, DEGs were selected by P<0.05, and cell types of clusters are first identified by singleR and Cell Markers database, then they are manually corrected by the markers in the original article. The different immune cells and non-immune cell expression matrices and metadata were imported into monocle2 (33). The high variable genes identified by monocle2 were used for pseudo-trajectory analysis to identify cell differentiation trajectories and identify dynamical expression genes. The dynamical expression heatmaps were shown by the plot-pseudotime-heatmap function. Then we imported the expression matrix and metadata into CellChat (34), CellChat provides a database of ligand receptors in humans and mice to identify cell-cell actions in single-cell datasets. We first identified the different numbers and strengths of cell interactions between immune cells and non-immune cells to find the main sources and outputs of different types of cells, and we used Euclidean distance and information flow to infer the conservative and specific cell-cell communication signal pathways and ligand-receptor pairs that mediate cell communication of cell groups(thresh<0.05).

### Cell culture and transfection

In DMEM conditions containing 10% FBS, cells from the Hela and Siha cell lines were cultured. We purchased si-IL1B and si-ITGA5 from GenePharma. The following is the corresponding assay sequence of IL1B and ITGA5: si-IL1B: 5’-GATGTCTGGTCCATATGAA-3’; si-ITGA5: 5’-ACGAACCTCTTCTGTGATGGA-3’. We obtained the pGL4.10-CST7 promoter plasmid from Obio Technologies, Inc. Lipofectamine 2000 was used for cell transfection.

### Cell phenotype assay

Cells (3000/well) were inoculated on 96-well plates for the MTT assay, and absorbance was recorded at various time intervals to gauge cell viability.

Cells (2000/well) were inoculated in 6-well plates for the clonogenic assay, fixed after 14 days, and stained with crystal violet.

Thirty thousand cells were inoculated in the top chamber (Corning) of the Transwell migration test using serum-free media to promote migration to the bottom chamber. Then, cells were added in transwell chambers with or without matrigel covered in the chambers for invasion andmigration detection, respectively. The cells were fixed and stained after 24 hours. Using NIH ImageJ software, migratory cells were counted in three randomly chosen fields in each well.

### RNA isolation and qRT-PCR enzyme chain reaction

Following the manufacturer’s instructions, we extracted total RNA using the RNeasy mini kit (Cat. #74,101, Qiagen). The amount of RNA was then measured, and cDNA was produced using the reverse transcription. Real-time reverse transcription-PCR (qRT-PCR) was used to evaluate the cDNA, and the iQ™ SYBR^®^ Green Super Mix (Bio-RAD) was used. The following were the primer sequences:

GAPDH:5’-GGAGCGAGATCCCTCCAAAAT-3’,5’-GGCTGTTGTCATACTTCTCATGG-3’.
IL1B: 5’-ATGATGGCTTATTACAGTGGCAA-3’, 5’-GTCGGAGATTCGTAGCTGGA-3’; ITGA5:5’-AGACATTCGATCCCTCTACAACT-3’, 5’-AATCGGCCAAACTCATCATGG-3’;
CST7: 5’-TTGTTCAAGGAGTCCCGCATC-3’, 5’-GTCACAGTCATCCAGACGCA-3’;

### Western blot analysis

After placing the cells in RIPA buffer (MilliporeSigma), which contains phosphatase inhibitor and protease inhibitor, and lysing them on ice for 30 minutes, the supernatant was collected by high-speed centrifugation. Protein extract samples were run through SDS-PAGE gels to separate them. They were then put on PVDF membranes, covered with 5% skim milk, and subjected to primary and secondary antibody incubation.

### Statistical analysis

The Wilcoxon test compared two sets of data, while the Kruskal-Wallis test was used when there are more than two data groups. The Kaplan-Meier curve was used to visualize the results of the survival analysis (log-rank test). Built heat maps using the “pheatmap” package. The link between the ICI score subgroups and the incidence of somatic mutations was examined using a chi-square test and Spearman correlation analysis. Further analysis of the results of the CIBERSORT method was found statistically meaningful with a P<0.05. Each analysis was performed using the R program (version 4.1.1).

## Result

### The current status of cervical cancer immune microenvironment

**Figure 1:**
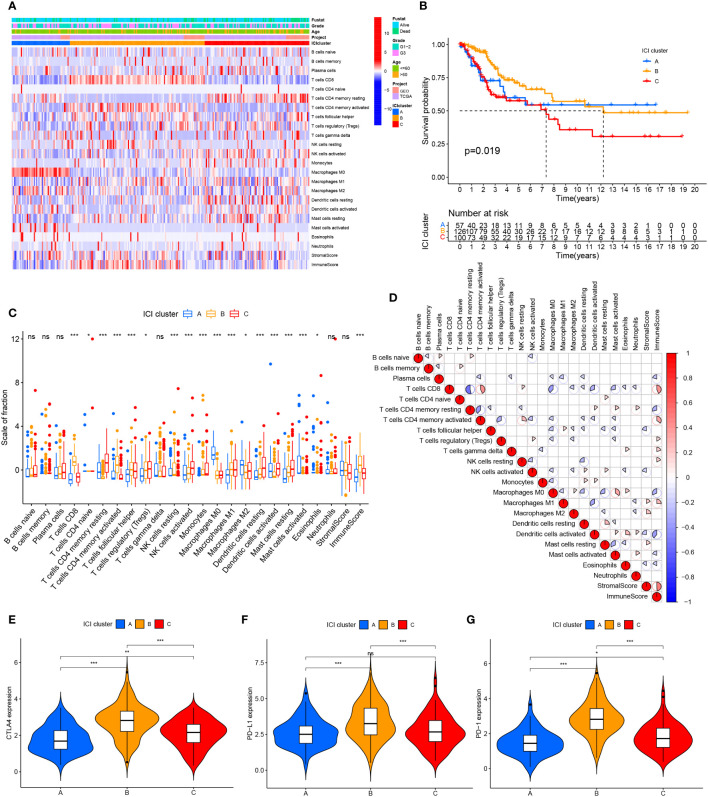
Cervical cancer tumor immune cell microenvironment landscape. **(A)** Used CIBERSORT algorithm unsupervised clustering of 22 immune cells on cervical cancer patients from TCGA and GEO databases. Clustered patients into three groups based on the degree of immune infiltration. Tumor immune infiltrating cells were represented by rows, and cervical cancer patient samples were represented by columns. **(B)** ICI clusters A, B, and C cervical cancer patients’ overall survival was shown using the Kaplan-Meier curve, log-rank test, P=0.019. **(C)** ICI clusters A, B, and C’s stromal scores, immune scores, and subgroups of 22 tumor immune cell infiltration levels are all included. **(D)** Intrinsic relationship between immunological score and tumor immune cell invasion. **(E–G)** differences in the three ICI clusters’ CTLA4, PD-L1, and PD-1 expression levels. ns, No significance, *P < 0.05, **P < 0.01, ***P < 0.001.

We searched public databases and merged 354 cervical cancer date from the TCGA and GEO databases. Based on the ESTIMATE and CIBERSORT algorithms, evaluated the immune cell subsets of cervical cancer and the immune characteristics of cervical cancer. According to the results of the CIBERSORT algorithm, unsupervised clustering of 22 immune cell types led to the discovery of three separate ICI clusters (**Supplementary Figure 1**; **Figure 1A**). The three separate ICI clusters’ overall survival rates were significantly different from one another(P=0.019) (**Figure 1B**); among them, the prognosis for Cluster B is the best, while Cluster C is the worst. To investigate potential relationships between immune cell infiltration and immune cell clusters, we examined the immune cell composition in three distinct clusters (**Figure 1C**). We discovered significant differences in immune scores between the three clusters but no differences in stromal scores. The relationship between the different immune cell infiltrates was shown using a heat map (**Figure 1D**). T cells CD8 and T cells CD4 memory activated demonstrated greater levels of activation in cluster B. In Cluster C, there are more Dendritic cells resting, NK cells activated, Mast cells resting, and T cells CD4 memory resting. Additionally, in order to more thoroughly examine the innate immunotherapy potential of various immune cell clusters, we analyzed the level of prominent immune checkpoints in these three ICI clusters. It showed that CTLA4 (**Figure 1E**), PD-L1 (**Figure 1F**), and PD-1 (**Figure 1G**) had higher expression levels in cluster B.

### Identification of differential expression of identified immune gene subtypes

**Figure 2:**
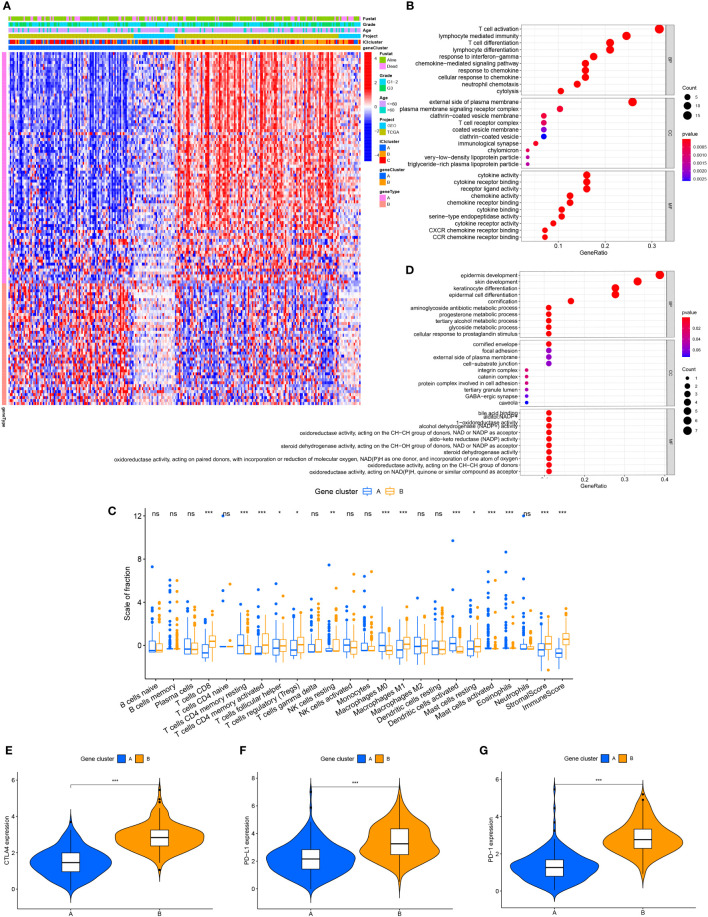
The production of immune internal genes and subtype classification. **(A)** Gene clusters A and B were used to separate patients into two categories using unsupervised clustering of shared DEGs generated from three ICI cluster groupings. **(B)** Gene ontology (GO) enrichment analysis of gene cluster A. **(C)** Gene ontology (GO) enrichment analysis of gene cluster B. **(D)** Tumor immune cell infiltration subgroups of gene cluster A and B, as well as stromal score and immune score. **(E–G)** The differences in CTLA4, PD-L1, and PD-1 expression between gene clusters A and B. ns, No significance, *P < 0.05, **P < 0.01, ***P < 0.001.

To compare the variations in transcriptional expression across the three distinct ICI clusters, we utilized the “limma” package to obtain 119 DEGs from 3 ICI clusters. Unsupervised clustering was used to separate the samples from the TCGA and GEO datasets into distinct gene groups, which were given gene cluster A and B (**Figure 2A**; **Supplementary Figure 2**), genes in gene cluster A are connected with characteristics in a positive way, whereas genes in gene cluster B are correlated with traits in a negative one. The Kaplan-Meier curve was used to compare the overall survival between the two gene clusters. There was no significant difference in the overall survival between the two gene clusters (P=0.441) (**Supplementary Figure 3A**). However, when comparing the median survival time between the two, we found that the median survival time was higher for gene cluster A. We performed GO enrichment analysis on two gene clusters, and it showed that gene cluster A was mainly enriched in immune cell activation and immune signal transmission pathway, and it was an important part of the cell transmembrane signal transmission process (**Figure 2B**). In gene cluster B, it showed strong enrichment characteristics during the development and differentiation of the epidermis and matrix (**Figure 2C**). The infiltration level of 22 immune cells in two gene clusters was examined using the CIBERSORT algorithm to learn more about the potential roles of different gene clusters in the immunological milieu (**Figure 2D**). The majority of the immune cells in gene cluster B have been discovered to have a higher active degree of immune infiltration. This implied that gene cluster B was in a state of immune activation. In addition, in gene cluster B, immune-related genes CTLA4, PD-L1, and PD-1 all had higher levels of expression (**Figures 2E–G**), which indicates that immunotherapy can exert a better effect in gene cluster B.

### Construction of ICI scoring system for cervical cancer

**Figure 3:**
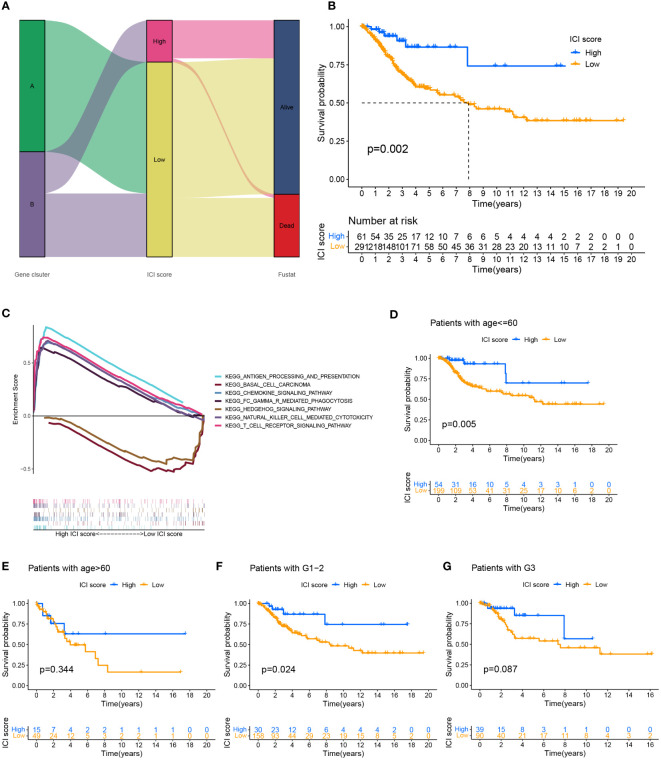
Establishment of ICI scoring system. **(A)** Used the Sankey chart to depict the correlation between two gene clusters, different ICI scores, and different living conditions. **(B)** Plotted the Kaplan-Meier curve (log-rank test) to display the two ICI score groups’ overall survival rates (P=0.002). **(C)** GSEA analysis of high and low ICI score groups associated with KEGG enrichment. Survival analysis of ICI score subgroups of different clinical traits. **(D, E)** The overall survival rate of cervical cancer patients of various ages in two ICI score groups was shown using the Kaplan-Meier curve. Patients with cervical cancer under the age of 60 showed higher overall survival in the group with high ICI scores (P=0.005). The overall survival rate for individuals older than 60 years old was not significantly different between the groups with high and low ICI scores (P=0.334). **(F, G)** Kaplan-Meier curve of cervical cancer patients in two ICI score groups with varying grades, log-rank test. High ICI scores among cervical cancer patients with low grades were associated with improved overall survival (P=0.024). There was no variation in the survival prognosis of high-grade patients across ICI score categories (P=0.087).

We performed a PCA analysis to quantify the combined factors of immune cell infiltration in cervical cancer. Recorded the sum of the individual scores of each sample as the ICI scores of two gene clusters to construct an ICI scoring system based on ICI phenotypic characteristics. Sankey diagrams depict the pattern of various gene clusters, ICI scores, and patient survival rates for cervical cancer (**Figure 3A**). When the survival prognosis of the high and low ICI score groups was compared using the Kaplan-Meier curve, the overall survival of the high ICI score group was greater (P=0.002) (**Figure 3B**). Used GSEA to analyze the function of the KEGG cell pathway, immune cell activation, immune cell killing and antigen presentation, and other immune cell working pathways were the main enriched pathways in the high ICI score group; growth of stromal cells was the main enrichment pathway in the low ICI score group (**Figure 3C**). To more effectively assess the ICI score’s effectiveness in predicting the prognosis of cervical cancer, we combined different clinical traits and performed a correlation analysis of the clinical traits between different ICI score groups (**Supplementary Figure 3B**), it showed that for patients with different grades, the patients with the worse grade had higher ICI scores (P=0.04). However, when we examined the relationship between various age groups and ICI scores, we discovered no statistically significant difference (P=0.23) (**Supplementary Figure 3C**). We also analyzed the survival prognosis in these two ICI score groups with different clinical characteristics. In patients under 60 years of age, overall survival was higher in patients with high ICI scores (p=0.005) (**Figure 3D**), and the same findings were also seen in the G1-2 patient group with high ICI scores (P=0.024) (**Figure 3F**). However, in the group of people older than 60 years (**Figure 3E**) and the G3 stage group (**Figure 3G**), there were no significant differences between the two ICI score groups.

### Construction of the correlation between tumor somatic mutation and ICI score

Somatic mutations often occur in nature; when the tumor mutation burden (TMB) increases, it can lead to the production of new antigens and induce anti-cancer responses in immune cells, which also provides opportunities for immunotherapy, high TMB suggests benefit for immunotherapy of tumors (35). Somatic mutations (SNVs) data for cervical cancer were obtained from the TCGA database to investigate the potential relationship between tumor mutation burden in the immune microenvironment of cervical cancer and ICI scores. Exploring the difference in TMB between the two ICI score groups revealed that the group with higher ICI score had a higher TMB index (p=0.0017) (**Supplementary Figure 4A**) and a positive correlation between the two variables (Spearman coefficient: R=0.19, P=0.0013) (**Supplementary Figure 4B**). In addition, overall survival was more satisfactory in the high mutation burden group compared to the low mutation burden group (P=0.042) (**Supplementary Figure 4C**). In order to do a stratified survival prognosis analysis, we integrated the ICI scores and TMB. The survival outcomes of these four different subgroups were significantly different (P=0.007) (**Supplementary Figure 4D**).

The “maftools” package was used to identify the top 20 genes most likely to be mutated in each ICI score group to determine the genes with the most significant mutation risk in each ICI score subgroup (**Supplementary Figures 4E, F**), including missense, nonsense. Additionally, we discussed a possible connection between TMB and ICI scores (**Table 1**).

**Table 1:** The internal connection between ICI score and mutation load.

gene	H-wild	H-mutation	L-wild	L-mutation	pvalue
CHD6	59 (85.51%)	10 (14.49%)	210 (96.77%)	7 (3.23%)	0.001602
BIRC6	60 (86.96%)	9 (13.04%)	210 (96.77%)	7 (3.23%)	0.005265
MXRA5	61 (88.41%)	8 (11.59%)	210 (96.77%)	7 (3.23%)	0.016122
F8	61 (88.41%)	8 (11.59%)	210 (96.77%)	7 (3.23%)	0.016122
PCNT	61 (88.41%)	8 (11.59%)	210 (96.77%)	7 (3.23%)	0.016122
PCLO	57 (82.61%)	12 (17.39%)	202 (93.09%)	15 (6.91%)	0.018435
DNAH3	61 (88.41%)	8 (11.59%)	209 (96.31%)	8 (3.69%)	0.0286
FLG2	61 (88.41%)	8 (11.59%)	209 (96.31%)	8 (3.69%)	0.0286
KMT2C	49 (71.01%)	20 (28.99%)	182 (83.87%)	35 (16.13%)	0.02889
MUC17	56 (81.16%)	13 (18.84%)	198 (91.24%)	19 (8.76%)	0.036119
UBR4	60 (86.96%)	9 (13.04%)	206 (94.93%)	11 (5.07%)	0.046428
DNAH9	61 (88.41%)	8 (11.59%)	208 (95.85%)	9 (4.15%)	0.046975

### The role of ICI score in predicting immunotherapy benefits

**Figure 4:**
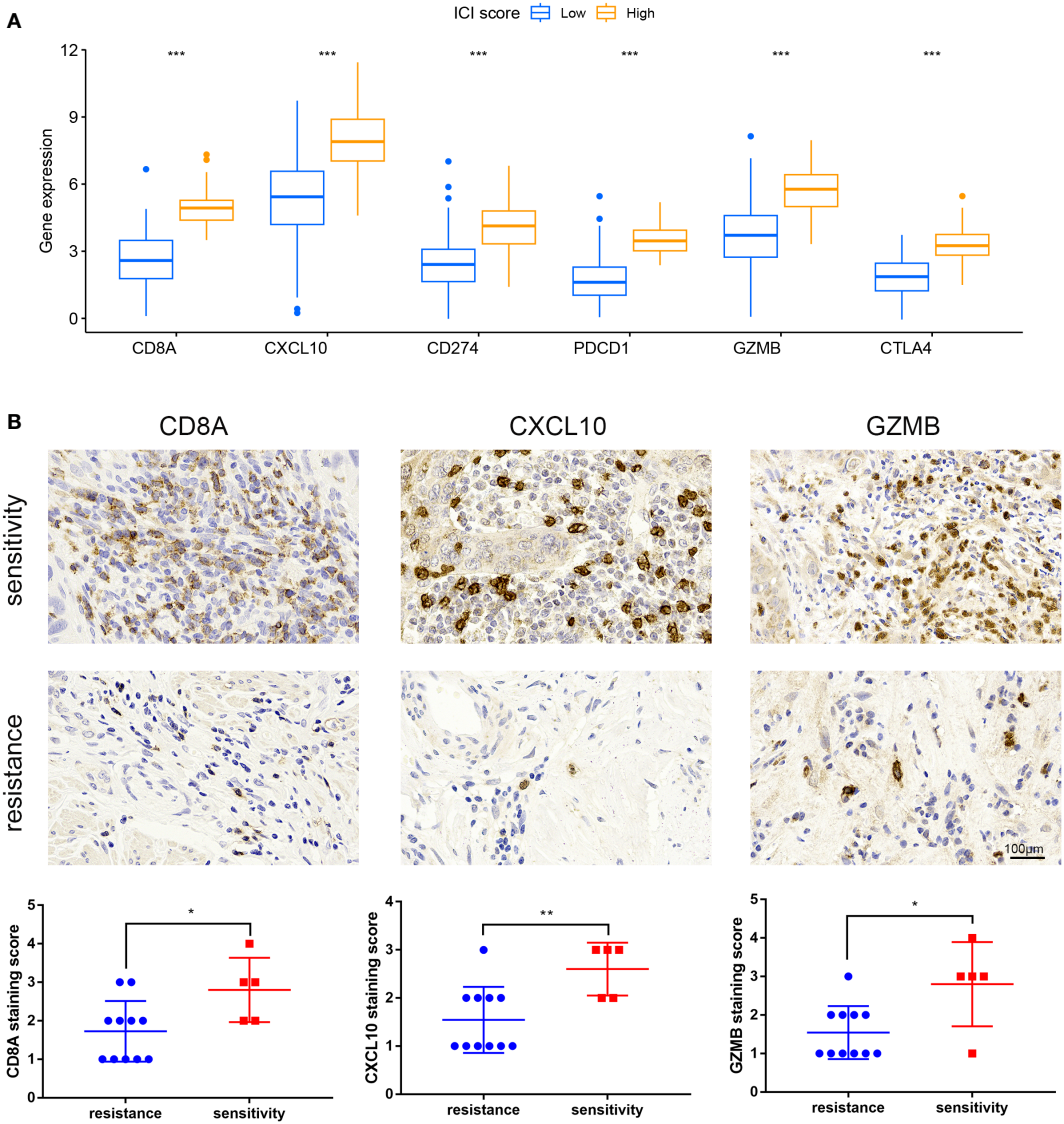
Expression of immune checkpoint and immunological activation-related genes and ICI score. **(A)** Expression levels of CD8A, CXCL10, CD274, PDCD1, GZMB, and CTLA4 were compared across groups with high and low ICI scores. **(B)** IHC staining of samples from our hospital to detect CD8A, CXCL10, GZMB expression. *P < 0.05, **P < 0.01, ***P < 0.001.

**Figure 5:**
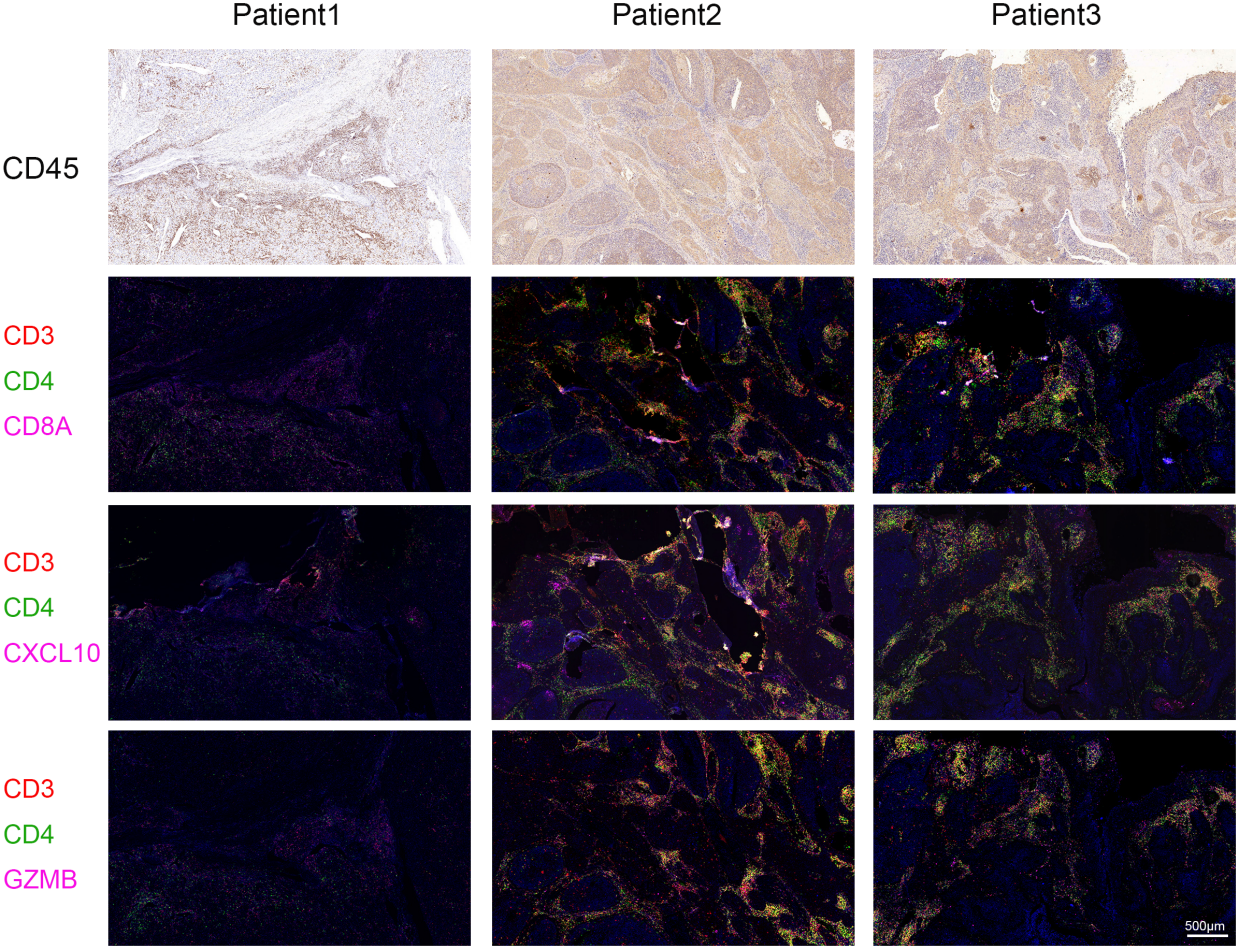
Multiplex Immunofluorescence. The expressions of CD8A, CXCL10 and GZMB were detected by mIHC staining in specimens of our hospital. Immunotherapy efficacy of patient1 was evaluated as resistant and immunotherapy efficacy of patient2 and patient3 was evaluated as sensitive. CD3 is represented by red fluorescence, CD4 by green fluorescence, and CD8A, CXCL10, and GZMB by pink fluorescence.

Immune checkpoint-related genes such as PDCD1(PD-1), GZMB, CD274(PD-L1), and CTLA4, as well as immune activation-related genes CD8A and CXCL10, were used as characteristic genes to assess the immune activity of the two ICI score groups. The expression levels of these genes were compared. The results revealed that the expression of all these genes was higher in the high ICI score group (**Figure 4A**). To investigate the potential correlation between ICI scores and immunotherapy, we included 16 cervical cancer patients who had received PD1, PDL1, and VEGF immunotherapy, and obtained cervical tissues from these patients prior to receiving immunotherapy. The Response Evaluation Criteriain Solid Tumours (RECIST) efficacy assessment method was used to evaluate the immunotherapy effect of these patients, among which 5 patients were sensitive to immunotherapy and 11 patients were resistant to immunotherapy. IHC results revealed that patients who responded well to immunotherapy had higher expressions of CD8A, CXCL10, and GZMB (**Figure 4B**). Next, we performed a further evaluation using mIHC, in which patient1 showed immunotherapy resistance and patient2 and patient3 showed immunotherapy sensitivity. It can be seen that in the immunotherapy-sensitive group, the expressions of CD45, CD3 and CD4 were all increased, in addition, the expressions of CD8A, CXCL10 and GZMB were also increased (**Figure 5**).

We evaluated the IC50 of Cisplatin, 5-Fluorouracil, Vinblastine, Vincristine, Paclitaxel, Docetaxel, Gemcitabine, Cyclophosphamide, Topotecan, Epirubicin, Olaparib, Dactinomycin in cervical cancer, and found that the drug sensitivity was higher in the high ICI score group (**Supplementary Figure 5**). This indicates that ICI score has great potential in predicting chemotherapy drug sensitivity.

### Identification of key genes and determination of gene function in cervical cancer

Univariate COX analysis was used to filter important genes from 119 DEGs, and 21 genes associated with the prognosis of cervical cancer were eliminated. Then, three important genes were found using the multivariate COX analysis, namely CST7, IL1B, and ITGA5 (**Supplementary Figure 6A**). These three key genes were used to build a prognostic model (**Supplementary Figures 6B–D**), and the results of the 1, 3, 5year ROC curves created using the model demonstrated that they could make accurate predictions (**Supplementary Figures 6E, F**).

**Figure 6:**
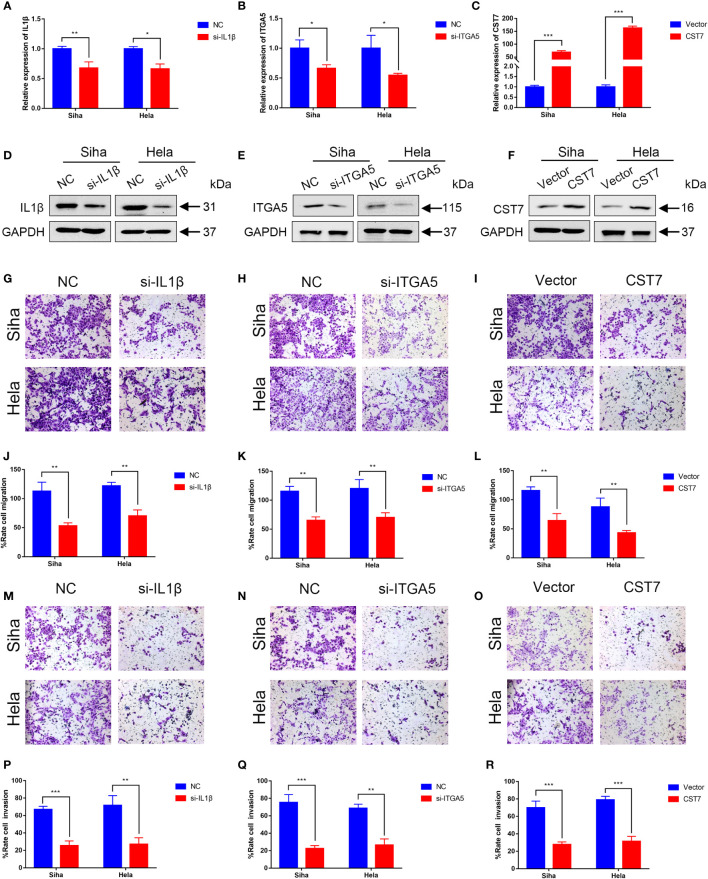
Validation of IL1B, ITGA5 and CST7 in cervical cancer cell lines. **(A–C)** The knockdown levels of IL1B and ITGA5 and the overexpression of CST7 in Siha and Hela cell lines were verified by qRT-PCR. **(D–F)** Western blot was utilized to confirm the overexpression of CST7 and the knockdown of IL1B and ITGA5 in Siha and Hela cell lines **(G–L)** Transwell migration tests were utilized to quantify the degree of cell migration in Siha and Hela cells after IL1B and ITGA5 were knocked down and CST7 was overexpressed, as well as to statistically evaluate the proportion of moved cells (n = 3). **(M–R)** Transwell migration tests were utilized to quantify the degree of cell invasion in Siha and Hela cells after IL1B and ITGA5 were knocked down and CST7 was overexpressed, as well as to statistically evaluate the proportion of moved cells (n = 3). *P < 0.05, **P < 0.01, ***P < 0.001.

**Figure 7:**
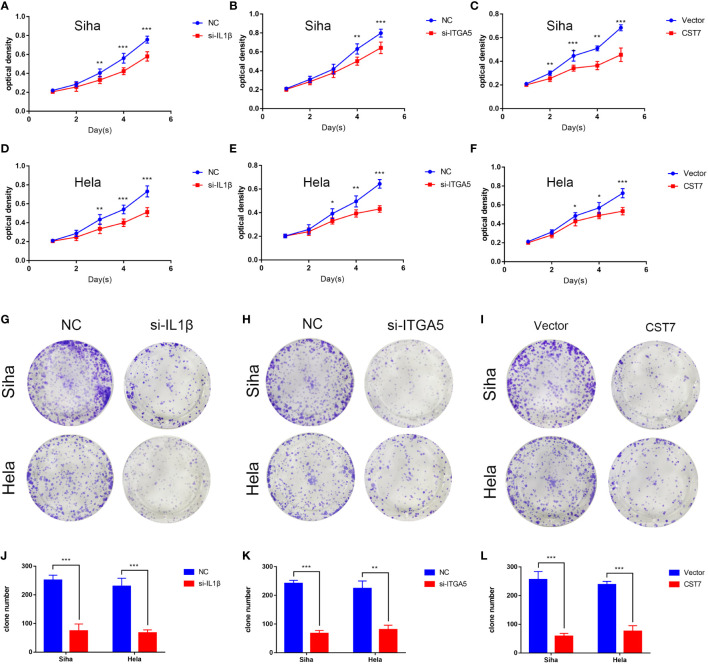
IL1B and ITGA5 knockdown and CST7 overexpression inhibit the proliferation of cervical cancer cells. **(A–F)** IL1B and ITGA5 knockdown and CST7 overexpression were assessed using the MTT test to assess the viability and proliferation of Siha and Hele cells (n = 3). **(G–L)** Cell proliferation after knockdown of IL1B and ITGA5 and overexpression of CST7 was determined by clonal assay. Statistical analysis of the colony-forming ability of Siha and Hele cells was performed (n = 3). *P < 0.05, **P < 0.01, ***P < 0.001.

We then performed cellular experiments to investigate the role of CST7, IL1B and ITGA5 in cervical cancer cells. We treated CST7 with plasmid overexpression and verified the knockdown efficiency of IL1B and ITGA5 and the overexpression efficiency of CST7 by qRT-PCR and western blot. The findings demonstrated that si-IL1B and si-ITGA5 had substantial knockdown efficiencies, and that CST7 overexpression was clearly present (**Figures 6A–F**). According to Transwell data, the capacity of cervical cancer cells to migrate was dramatically reduced when IL1B and ITGA5 were knocked down and CST7 was overexpressed in comparison to the control group (**Figures 6G–L**), similarly, the invasive ability of cervical cancer cells was significantly down-regulated (**Figures 6M–R**). MTT results showed that knockdown of IL1B and ITGA5 and overexpression of CST7 significantly reduced the proliferation of cervical cancer cells (**Figures 7A–F).** Additionally, the findings of the clonogenic assay demonstrated that overexpressing CST7 and knocking down IL1B and ITGA5 decreased the capacity of cervical cancer cells to proliferate (**Figures 7G–L**).

### Immune cell potential value of key genes

**Figure 8:**
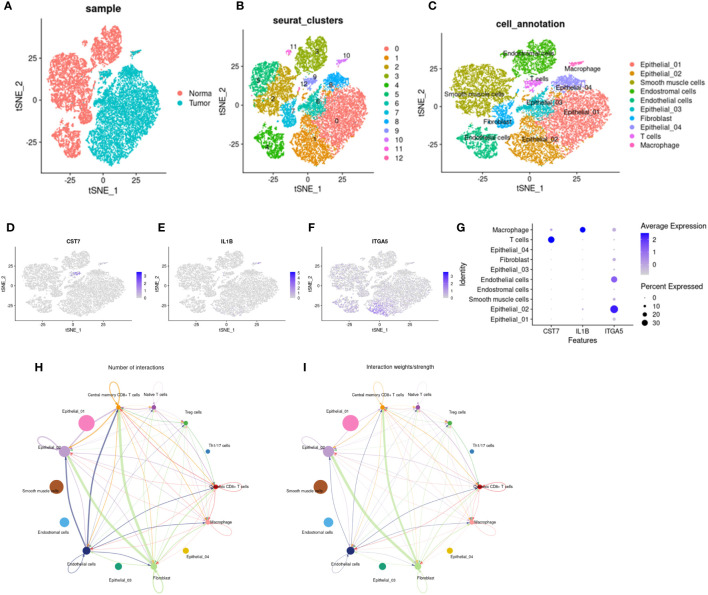
Distribution and cell communication of IL1B, CST7 and ITGA5 in immune cells. **(A)** Distribution of 24,499 cells in tumor tissue and normal tissue, with 11,394 cells in normal tissue and 13,104 cells in tumor tissue. **(B)** Cells were divided into 13 clusters by unsupervised clustering. **(C)** Cells were divided into 7 types based on cell-specific marker genes, including epithelial cells, smooth muscle cells, fibroblasts, endothelial cells, endometrial stromal cells, T cells, and macrophages. **(D–F)** Distribution of IL1B, CST7 and ITGA5 on different cell types. **(G)** Expression levels of IL1B, CST7 and ITGA5 on different cell types. **(H)** Communication pathways between cells distributed by IL1B, CST7 and ITGA5 **(I)** intensity of cell communication pathways.

To explore the potential role of IL1b, CST7, and ITGA5 in the immune microenvironment, we downloaded single-cell data of cervical cancer in GEO to validate our results and explore a more precise transcriptional landscape. The data set GSE168652 includes two samples, one from a tumor and one from adjacent normal tissue. After filtering low-quality cells, a total of 24498 cells (Normal: 11394; Tumor: 13104) remained to participate in downstream analysis. We used t-distributed stochastic neighbor embedding (t-SNE) to visualize the global distribution of cells (**Figures 8A–C**). Tumor tissue and normal tissue showed heterogeneity, with a distinct distribution of cells of different origins (**Figure 8A**). On this basis, unsupervised cluster analysis identified 13 clusters, and then we divided the cells into 7 main cell types according to the differentially expressed genes of each cluster and the cell-specific marker genes provided by the original article, including epithelial cells, smooth muscle cells, fibroblasts, endothelial cells, endometrial stromal cells, t cells and macrophages (**Figures 8B, C**). Then we showed the expression levels of these three key genes, CTS7, IL1B, and ITGA5, in different types of cells on the map (**Figures 8D–G**). All three genes were indeed expressed in immune cells. In particular, CST7 is highly expressed in T cells and IL1B is highly expressed in macrophages. Interestingly, ITGA5 is more widely expressed in non-immune cells, such as endothelial and epithelial cells. However, the expression of ITGA5 is concentrated in the epithelial subgroup Epithelial_02, suggesting that there may be some underlying knowledge in this cluster. The analysis of cell communication also shows that there are frequent interactions among cells expressing these three genes (**Figures 8H, I**).

**Figure 9:**
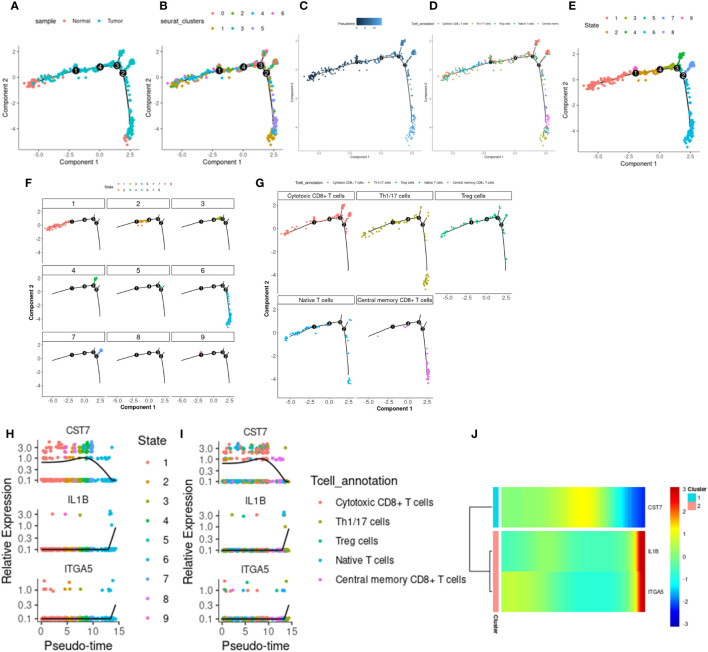
T cell differentiation track. **(A)** The distribution of tumor and normal cells in the T cell differentiation locus, which produced four differentiation nodes. **(B)** Branches generated by T cell differentiation locus, 7 branches in total. **(C)** The time distribution along the differentiation trajectory. The darker the color, the earlier the differentiation time. **(D)** Distribution of immune cells along the T cell differentiation trail. **(E)** Branches generated by immune cell differentiation in the T cell differentiation locus, with a total of 9 branches. **(F)** The corresponding cell positions of the nine branches on the differentiation trajectory. **(G)** cytotoxic CD8+ T cells, helper T cells, regulatory T cells, naive T cells, and central memory corresponding differentiation trajectory of CD8+ T cells. **(H)** Expression levels of IL1B, CST7 and ITGA5 in 9 branch cells. **(I)** IL1B, CST7, ITGA5 in cytotoxic CD8+ T cells, helper T cells, regulatory T cells, naive T cells, and central memory on CD8+ T cells. **(J)** Heat maps of expression patterns of IL1B, CST7 and ITGA5 on T cell differentiation trajectories.

We use monocle2 to explore the temporal order of immune cells and epithelial cells. We further subdivided T cells into 5 T cell subtypes according to the differentially expressed genes and classical marker genes of T cell subsets, including cytotoxic CD8+ T cells, helper T cells, regulatory T cells, naive T cells, and central memory CD8+ T cells. Four nodes and nine branches appeared on the pseudo-trajectories of T cells. Consistent with the results of pseudo-trajectories analysis, naive T cells first appeared and mainly gathered in state1, differentiated into other T cell subtypes, besides, helper T cells and regulatory T cells interacted with cytotoxic T cells along pseudo-trajectories (**Figures 9A–G**). However, macrophages show three cell states and differentiate from one starting point to two directions (**Supplementary Figures 7A–E**), which is consistent with our known knowledge. Under the influence of various cytokines, macrophages are polarized into M1/M2 macrophages, M1 macrophages are mainly involved in pro-inflammatory responses, and M2 macrophages are mainly involved in anti-inflammatory responses (36–38). Epithelial cells in tumor tissues exhibit complex diversity. We extracted Epithelial_02, a subpopulation highly expressing ITGA5, to reconstruct differentiation trajectories (**Supplementary Figures 7H–J**). Epithelial_02 is a malignant tumor cell that highly expresses some genes that promote tumor migration, invasion, and metastasis, such as the S100 family and CEACAM family genes (39–41). The expression patterns of 3 key genes were different in the pseudo-trajectories of the 3 types of cells, but IL1B and ITGA5 were more similar (**Figures 9H–J**; **Supplementary Figures 7F–G**, **Supplementary Figures 7K–L**), suggesting that CST7 and the other two work in a different mode of action in cancer.

## Discussion

The tumor immune microenvironment is a complex state, a suppressive tumor immune microenvironment is created due to specific immune cell infiltration, which is hypothesized to be connected to immunotherapy resistance and tumor growth (42). When tumorigenesis occurs, the organism triggers an immune response. When the balance between immunosurveillance and immunosuppressive cells in the tumor microenvironment is tilted, the body eventually loses immune surveillance of tumor cells, which promotes tumor progression (43).

Immunotherapeutic systems, such as immune checkpoint inhibitors, modified T cells like CAR T and T cell receptor (TCR) T cells, and other recently discovered immunotherapeutic methods, such as lysing viruses and bispecific antibodies, have evolved fast in recent years (44). In cervical cancer, immunotherapy has increasingly become a hot topic for researchers (45–47), immunotherapy is more customized than surgical intervention, neoadjuvant therapy, radiation, and chemotherapy. Immune checkpoint inhibitor use is crucial in the treatment of cervical cancer; for example, PD-1 and PD-L1 inhibitors can effectively benefit patients with cervical cancer (48–50). Protein kinase, DNA-activated, catalytic polypeptide (PRKDC) has a high mutational burden in cervical cancer (51), PRKDC has the function of repairing cervical cancer DNA, blocking the PRKDC pathway can effectively block the occurrence of this process, thereby increasing the sensitivity of chemotherapeutics (52). Studies have also found that the susceptibility of cervical cancer may be related to the polymorphisms of CTLA4 and IL-1 (53, 54). Additionally, the creation of immune cells by HPV to bypass host T cell monitoring and escape detection is linked to the development of cervical cancer (55, 56), immune escape clearance can effectively prevent and treat HPV persistent infection (57). Cervical cancer has a complex immune microenvironment. The chemotherapy process will activate local immune cells. The combined immune checkpoint inhibitor may become a new treatment direction (58–60).

In this study, we obtained cervical cancer patient information from the TCGA and GEO databases, assessed the immune microenvironment of cervical cancer, and divided the immune cells into three clusters, between various immune cells, it was shown that the correlation between active CD8+ T cells and CD4+ T cells was strongest. T cell activity and the expression levels of CTLA4, PD-1, and PD-L1 were highest in cluster B. Similarly, the survival prognosis of cluster B was also the best, which was consistent with previous research results (61–63). We obtained differential genes from three immune cell clusters and defined two gene clusters related to immune activation. The two gene clusters had differences in immune cell activation and matrix maturation. Additionally, we developed an ICI score network to present clinical characteristics, gauge the effectiveness of immunotherapy, and gauge the overall survival status of cervical cancer patients. The high ICI score group played an important role in immune cell activation, immune cell killing, antigen presentation and other processes. Elevated TMB levels can mediate immune cell activation, thereby benefiting immunotherapy (64, 65), we combined TMB and ICI scores in order to look into any possible association between them. The TMB and ICI scores showed a statistically significant positive connection. When comparing the overall survival rates of the various TMB subgroups, it was discovered that the high tumor mutation burden group had a higher prognosis for survival, which was in line with earlier research (66, 67). We examined the immune checkpoint- and immunological activation-related genes CXCL10, GZMB, CD8A, PD-1, PD-L1, and CTLA4 to evaluate the association between IC score and immunotherapy. The statistics show that the high ICI score group had greater expression of these genes. Therefore, we obtained tissues of cervical cancer patients from the hospital, and it was found that CXCL10, GZMB and CD8A were increased in patients sensitive to the efficacy of immunotherapy, suggesting that CXCL10, GZMB and CD8A may be potential therapeutic targets for cervical cancer immunotherapy. Drug sensitivity was correspondingly higher in subgroups with high ICI scores, according to the examination of chemotherapeutic drug sensitivity. We also identified 3 key genes, including IL1B, ITGA5 and CST7, from 119 differential genes. IL1B may encourage the development of tumor cells. By inhibiting IL1B, one may counteract breast cancer’s immunosuppressive effects and boost their anti-PD-1 counterparts, resulting in an anti-tumor impact (68). IL1B can also enhance the signal transduction of CTLA4, and targeting the IL1B-CTLA4 axis can effectively inhibit the occurrence of metastatic and recurrent colon cancer (69). ITGA5 is a crucial immunotherapy predictive target that is thought to be crucial in the incidence and progression of many cancers (70–72). CST7 is associated with activation of CD4T and CD8T cells in liver cancer (73). In our study, IL1B and ITGA were up-regulated in cervical cancer, while CST7 was down-regulated in cervical cancer, we conducted cell experiments on 3 key genes, and the results showed that down-regulation of IL1B and ITGA5 and overexpression of CST7 could reduce the growth and migration ability of cervical cancer cells. Analysis of single cell sequencing data showed that CST7 and IL1B were mainly distributed in immune cells, among which CST7 was distributed in T cells, IL1B was distributed in macrophages, and ITGA5 was mainly expressed in epithelial cells.

In our study, immune cells in the subgroup with high ICI scores were in an activated state, the expression of several immune checkpoints was elevated, the high expression of immune checkpoints was consistent with immunotherapy sensitivity. Three key genes were identified from 119 DGEs of ICI score, and cell experiments confirmed that Key genes had an impact on the proliferation and migration ability of cervical cancer cells, moreover, single-cell sequencing data suggested that CST7 and IL1B were mainly distributed in immune cells, which suggested the potential important role of CST7 and IL1B in the immune microenvironment of cervical cancer. Therefore, we think that the ICI score is one of the predictors of cervical cancer survival and prognosis and that including the ICI score as a separate predictor of immunotherapy for cervical cancer is a project that might be investigated in the future.

However, research has a certain degree of limitations. The research data came from public database platforms. We also need more clinical research data to support the predictive value of ICI scores.

## Data availability statement

The datasets presented in this study can be found in online repositories. The names of the repository/repositories and accession number(s) can be found in the article/**Supplementary Material**.

## Ethics statement

The Zhongnan Hospital of Wuhan University ethical committee approved the research (ethics number: 2020029). Written informed consent for participation was not required for this study in accordance with the national legislation and the institutional requirements.

## Author contributions

SY, LZ, and SC led and supervised the study as co-authors. LZ, HW, and YG performed the public dataset collection, bioinformatics analysis, and generated graphs and tables; SY and SC performed the experiments; SY and LZ collected the tumor samples. SY and SC wrote the manuscript, and HC, MD, and N-YS revised the manuscript. All authors contributed to the article and approved the submitted version.

## Supplementary material

Supplementary Figure 1 Unsupervised clustering of immune infiltration of cervical cancer. **(A-H)** The matrix heat map of cervical cancer samples when K=2-9, the darker the color in the group, the higher the homogeneity in the group. The fewer the blank parts between groups, the more obvious the difference between the groups. **(I)** The unsupervised clustering area under the curve for K=2–9. **(J)** Based on the cumulative distribution consensus index of tumor immune cell infiltration, K=2-9.

Supplementary Figure 2 Co-identification of unsupervised clustering differential genes based on ICI score. **(A-H)** Co-identification clustering heat map when K=2-9. **(I)** The unsupervised clustering area under the curve for K=2–9. **(J)** According to the cumulative distribution consistency index of tumor immune cell infiltration, K=2-9.

Supplementary Figure 3
**(A)** Compared the survival prospects of gene clusters A and B using the Kaplan-Meier curve, P=0.441. **(B)** The correlation between grades and ICI scores shows that the better the grade, the higher the ICI score (P=0.04). **(C)**There was no correlation between different age groups and ICI scores(P=0.23).

Supplementary Figure 4 Correlation between tumor mutation burden and ICI score. **(A)** Tumor mutation burden between different ICI score groups(P=0.0017). **(B)**Used a scatter plot to display the relationship between ICI score and TMB, which revealed a favorable relationship (R=0.19, P=0.0013). **(C)** Kaplan-Meier curves for cervical cancer overall survival in the high-TMB and low-TMB groups, the overall survival of the high TMB group was better (P=0.042). **(D)** The overall survival rate of the stratified combination of TMB and ICI scores was shown using the Kaplan-Meier curve. **(E-F)** OncoPrint of the high ICI score group (left) and the low ICI score group (right), the samples were displayed on the horizontal axis, and the genes were displayed on the vertical axis. The filled colors represented different types of mutations.

Supplementary Figure 5 Correlation between risk score and chemotherapy drug response. Difference between chemotherapeutic drug IC50 between the high ICI score group and the low ICI score group.

Supplementary Figure 6 Construction of key genes risk model. **(A)** Multifactorial analysis was used to evaluate three important genes. **(B)** the distribution of cervical cancer patients’ risk indices. **(C)** The survival status chart of cervical cancer. **(D)** The heatmap of three key genes. **(E)** Examination of the risk model survival curve. **(F)** ROC curve to verify the ability of the risk model to predict prognosis.

Supplementary Figure 7 Differentiation trajectories of macrophages and epithelial cells. **(A)** The distribution of normal and tumor cells along the macrophage differentiation trail. One differentiation node is generated. **(B)** The time distribution on the differentiation trajectory of macrophages. The darker the color, the earlier the differentiation time. **(C)** Branches generated by macrophage differentiation locus, including 3 branches. **(D)** The distribution of tumor and normal cells on three branches. **(E)** Corresponding differentiation positions of cells on the three branches. **(F)** Expression levels of IL1B, CST7 and ITGA5 in three branch cells. **(G)** Heat maps of expression patterns of IL1B, CST7 and ITGA5 on macrophage differentiation trajectories. **(H)** The time distribution of epithelial cell differentiation tracks, the darker the color, the earlier the differentiation. **(I)** The branching of epithelial cells along the differentiation path, with a total of 7 branches. **(J)** The corresponding position of cells on each branch of the epithelial differentiation pathway. **(K)** Expression levels of IL1B, CST7 and ITGA5 in 7 branch cells. **(L)** Heat map of expression patterns of IL1B, CST7 and ITGA5 in epithelial cell differentiation tracks.

## Glossary

**Table:**

HPV	Human papilloma virus
FDA	Food and Drug Administration
IL-2	The interleukin-2
CTLA-4	Cytotoxic T lymphocyte-associated protein 4
PD-1	Programmed cell death 1
PD-L1	Programmed cell death-Ligand 1
TCGA	The Cancer Genome Atlas
GEO	The Gene Expression Omnibus
ICI	Immune cell infiltration
PCA	Principal component analysis
GO	Gene ontology
GSEA	Gene Set Enrichment Analysis
GDSC	The Genomics of Drug Sensitivity in Cancer
IC50	The drug’s predicted half-maximal inhibitory concentration
CD8A	CD8 antigen, alpha chain
CXCL10	C-X-C motif chemokine ligand 10
GZMB	Granzyme B
BSA	Bovine serum albumin
CD3	CD3 antigen, epsilon polypeptide
CD4	CD4 Molecule
CST7	Cystatin F
IL1B	Interleukin 1 beta
ITGA5	Integrin subunit alpha 5
DEGs	Differentially expressed genes
