# Supplementary figures and images for: Cervical cancer immune infiltration microenvironment identification, construction of immune scores, assisting patient prognosis and immunotherapy
Source: Front Immunol. 2023 Mar 10;14:1135657. doi: 10.3389/fimmu.2023.1135657 (PMC10037308; doi:10.3389/fimmu.2023.1135657)

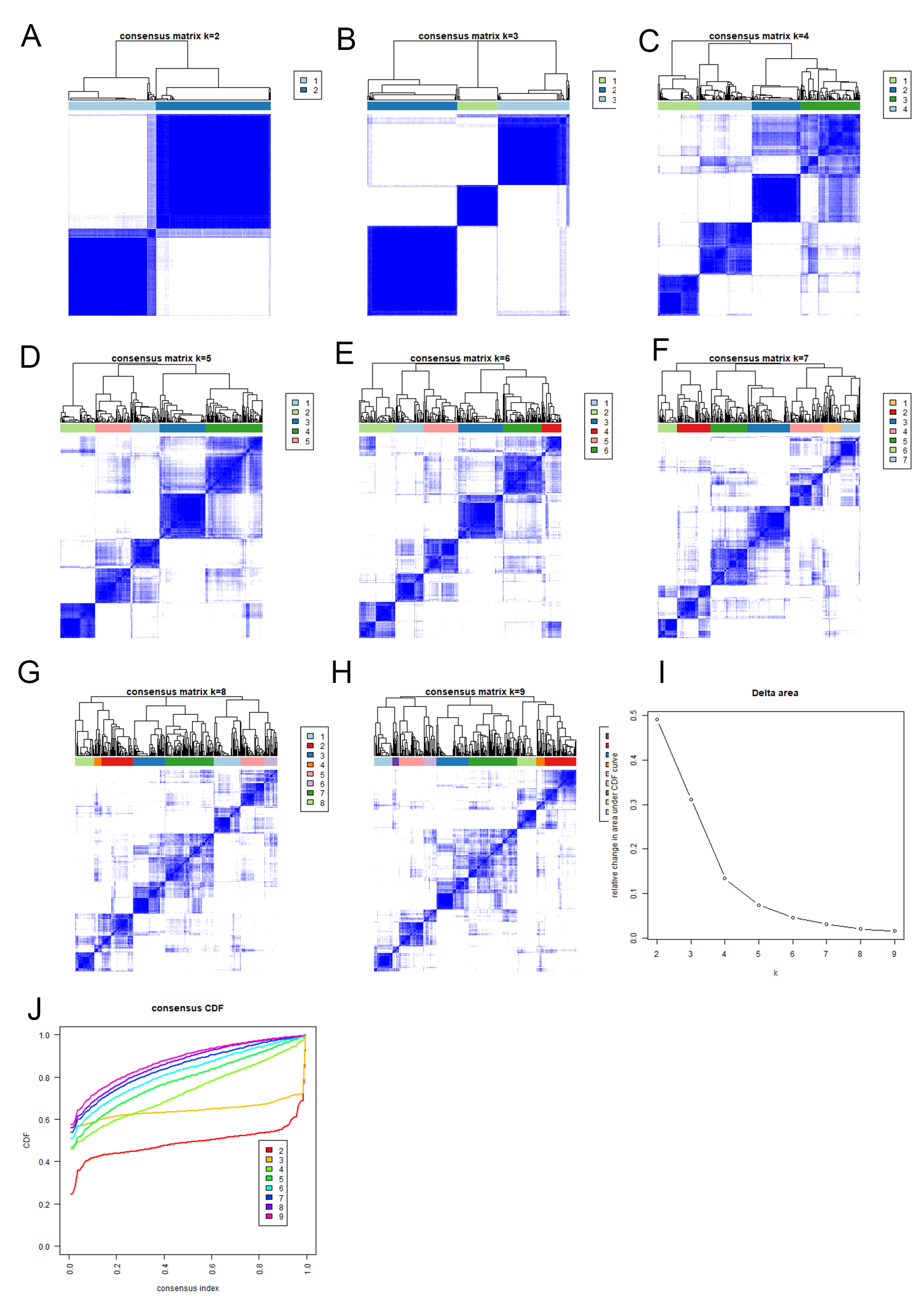

Supplement: Supplementary Figure 1 — Unsupervised clustering of immune infiltration of cervical cancer. (A-H) The matrix heat map of cervical cancer samples when K=2-9, the darker the color in the group, the higher the homogeneity in the group. The fewer the blank parts between groups, the more obvious the difference between the groups. (I) The unsupervised clustering area under the curve for K=2–9. (J) Based on the cumulative distribution consensus index of tumor immune cell infiltration, K=2-9. [file Image_1.tif]

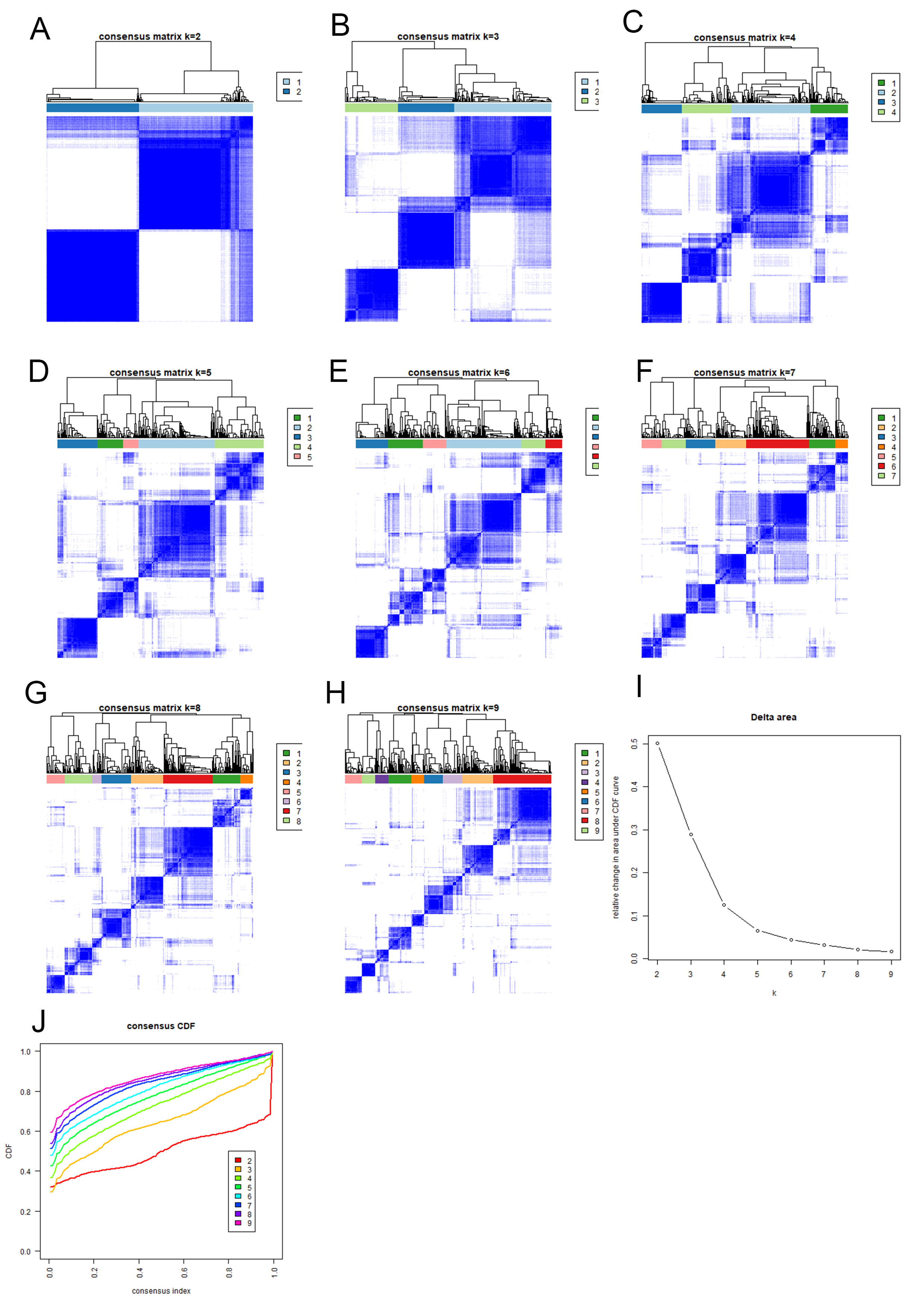

Supplement: Supplementary Figure 2 — Co-identification of unsupervised clustering differential genes based on ICI score. (A-H) Co-identification clustering heat map when K=2-9. (I) The unsupervised clustering area under the curve for K=2–9. (J) According to the cumulative distribution consistency index of tumor immune cell infiltration, K=2-9. [file Image_2.tif]

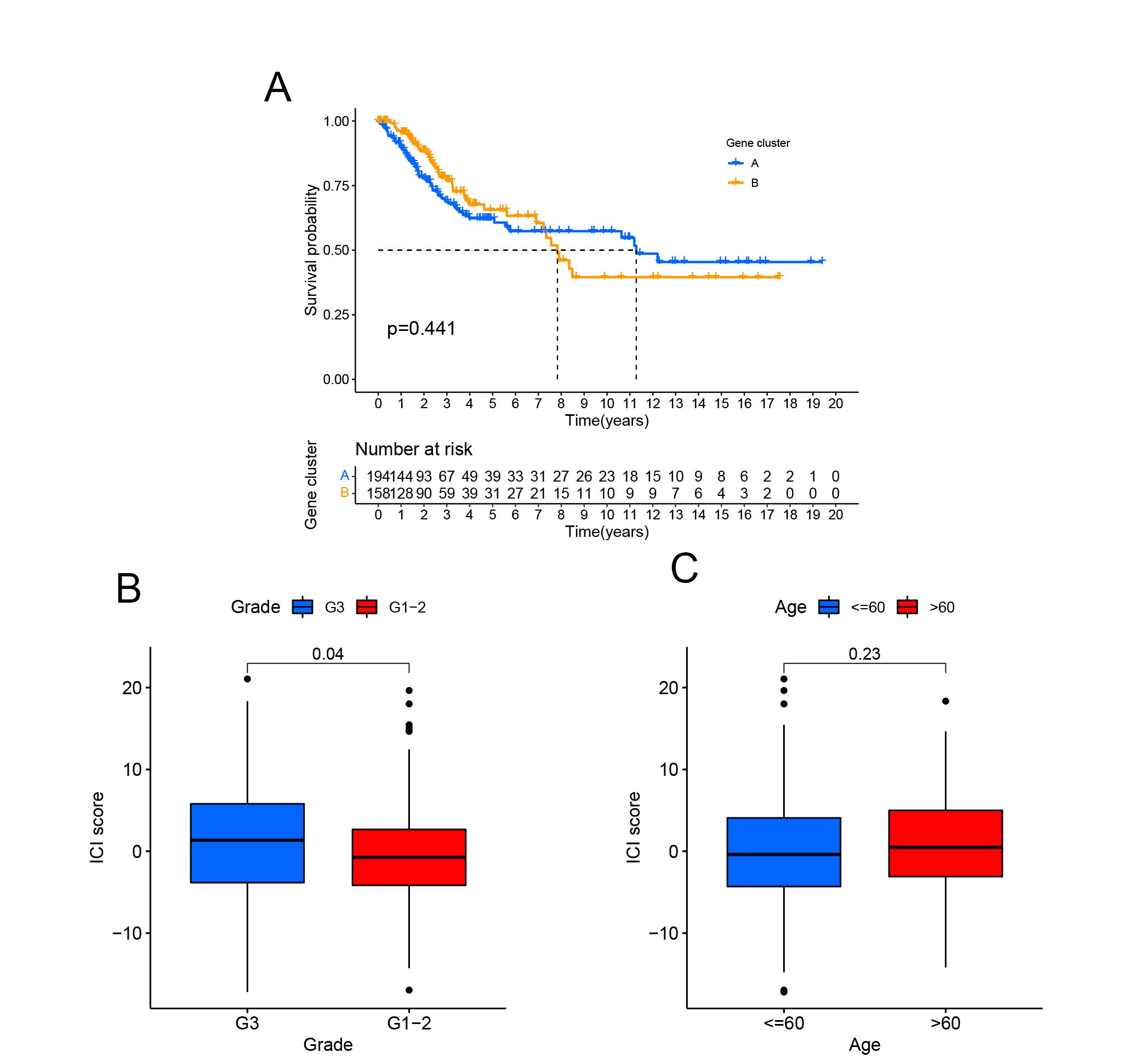

Supplement: Supplementary Figure 3 — (A) Compared the survival prospects of gene clusters A and B using the Kaplan-Meier curve, P=0.441. (B) The correlation between grades and ICI scores shows that the better the grade, the higher the ICI score (P=0.04). (C)There was no correlation between different age groups and ICI scores(P=0.23). [file Image_3.tif]

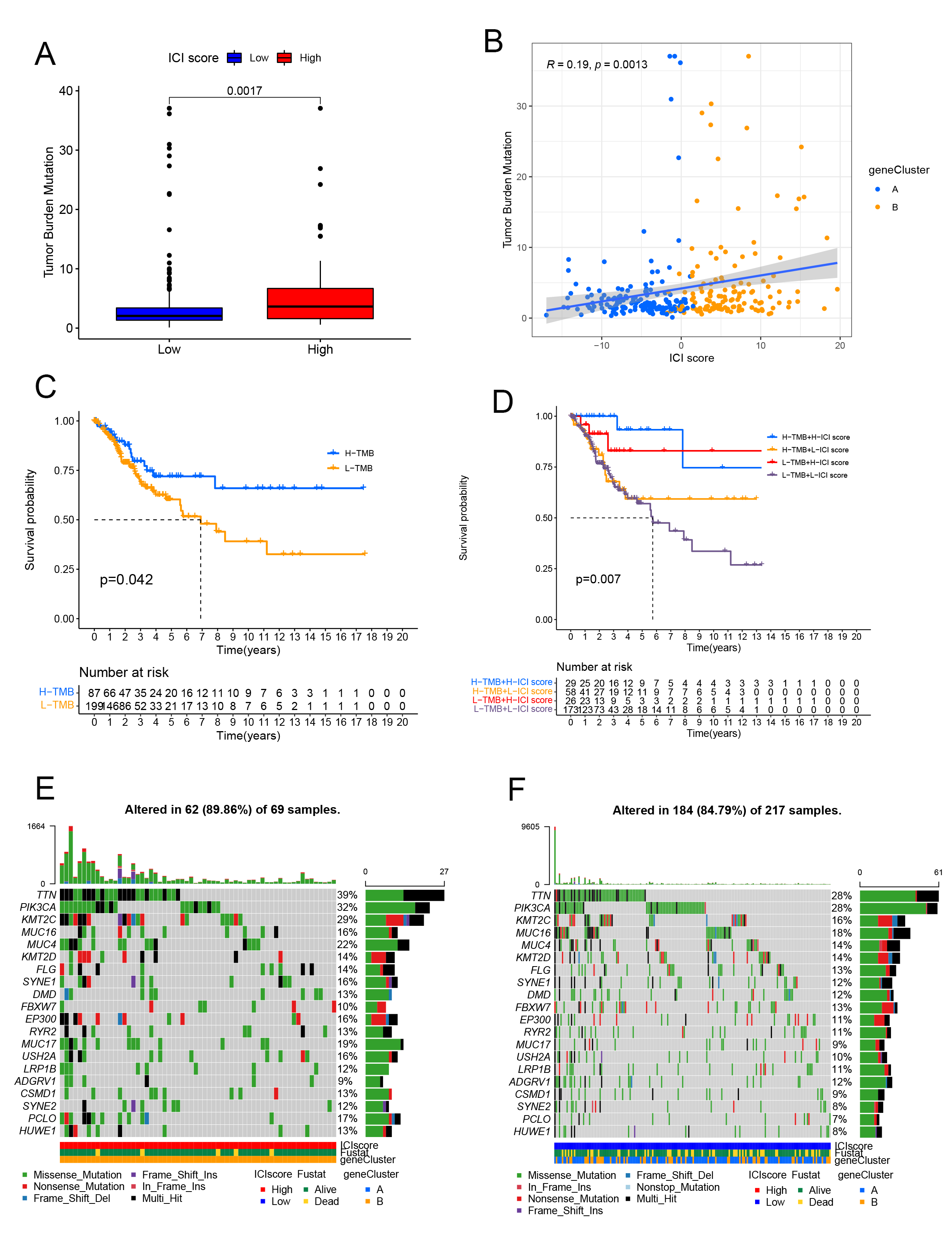

Supplement: Supplementary Figure 4 — Correlation between tumor mutation burden and ICI score. (A) Tumor mutation burden between different ICI score groups(P=0.0017). (B)Used a scatter plot to display the relationship between ICI score and TMB, which revealed a favorable relationship (R=0.19, P=0.0013). (C) Kaplan-Meier curves for cervical cancer overall survival in the high-TMB and low-TMB groups, the overall survival of the high TMB group was better (P=0.042). (D) The overall survival rate of the stratified combination of TMB and ICI scores was shown using the Kaplan-Meier curve. (E-F) OncoPrint of the high ICI score group (left) and the low ICI score group (right), the samples were displayed on the horizontal axis, and the genes were displayed on the vertical axis. The filled colors represented different types of mutations. [file Image_4.tif]

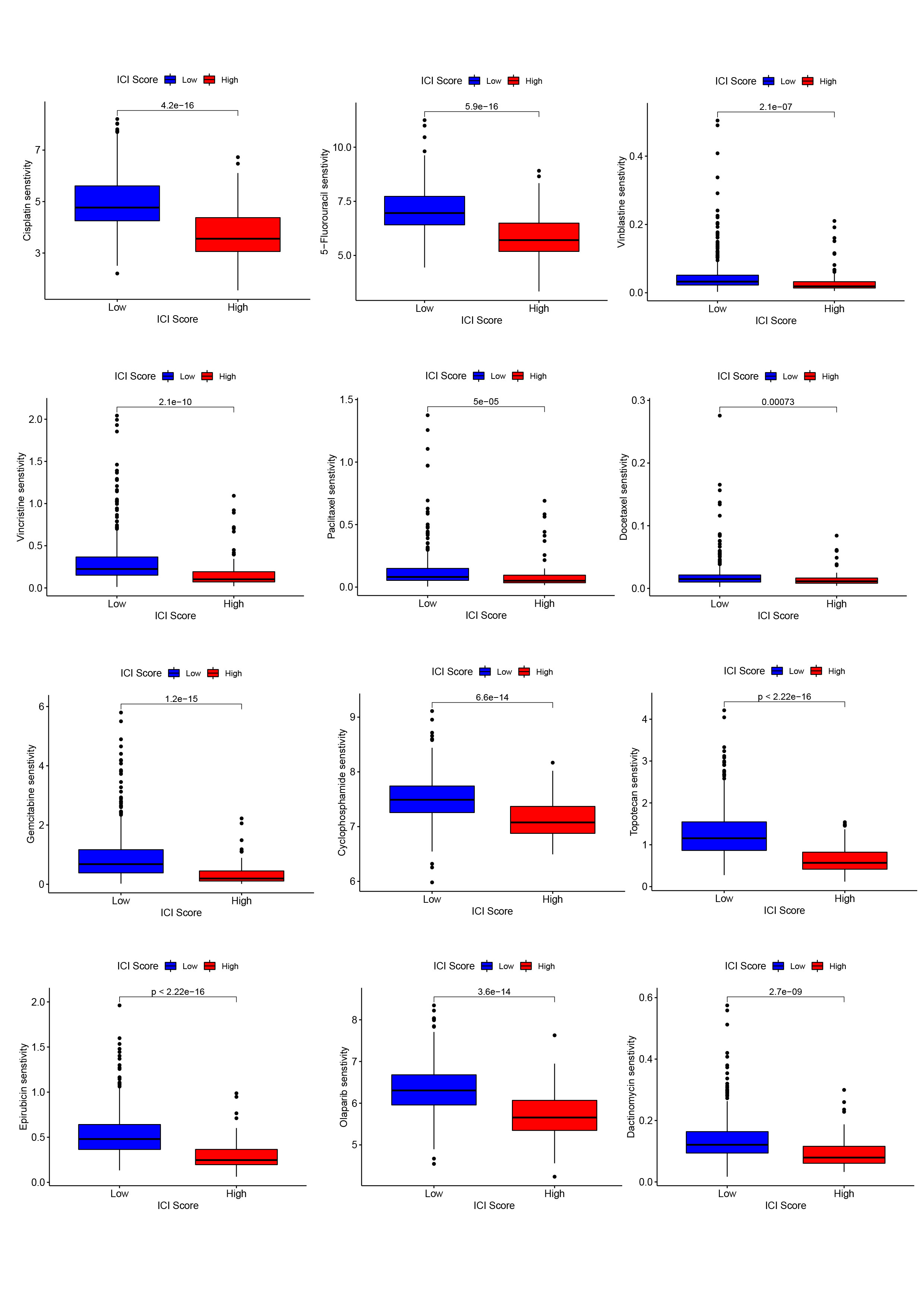

Supplement: Supplementary Figure 5 — Correlation between risk score and chemotherapy drug response. Difference between chemotherapeutic drug IC50 between the high ICI score group and the low ICI score group. [file Image_5.tif]

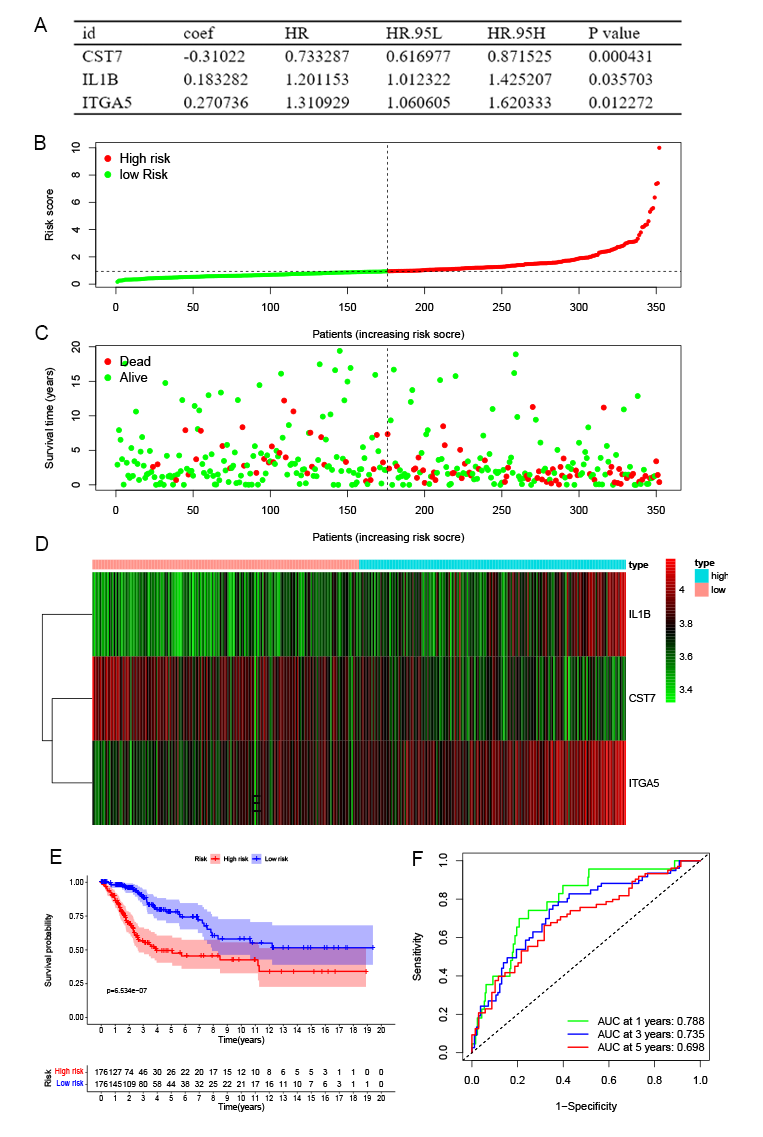

Supplement: Supplementary Figure 6 — Construction of key genes risk model. (A) Multifactorial analysis was used to evaluate three important genes. (B) the distribution of cervical cancer patients’ risk indices. (C) The survival status chart of cervical cancer. (D) The heatmap of three key genes. (E) Examination of the risk model survival curve. (F) ROC curve to verify the ability of the risk model to predict prognosis. [file Image_6.tif]

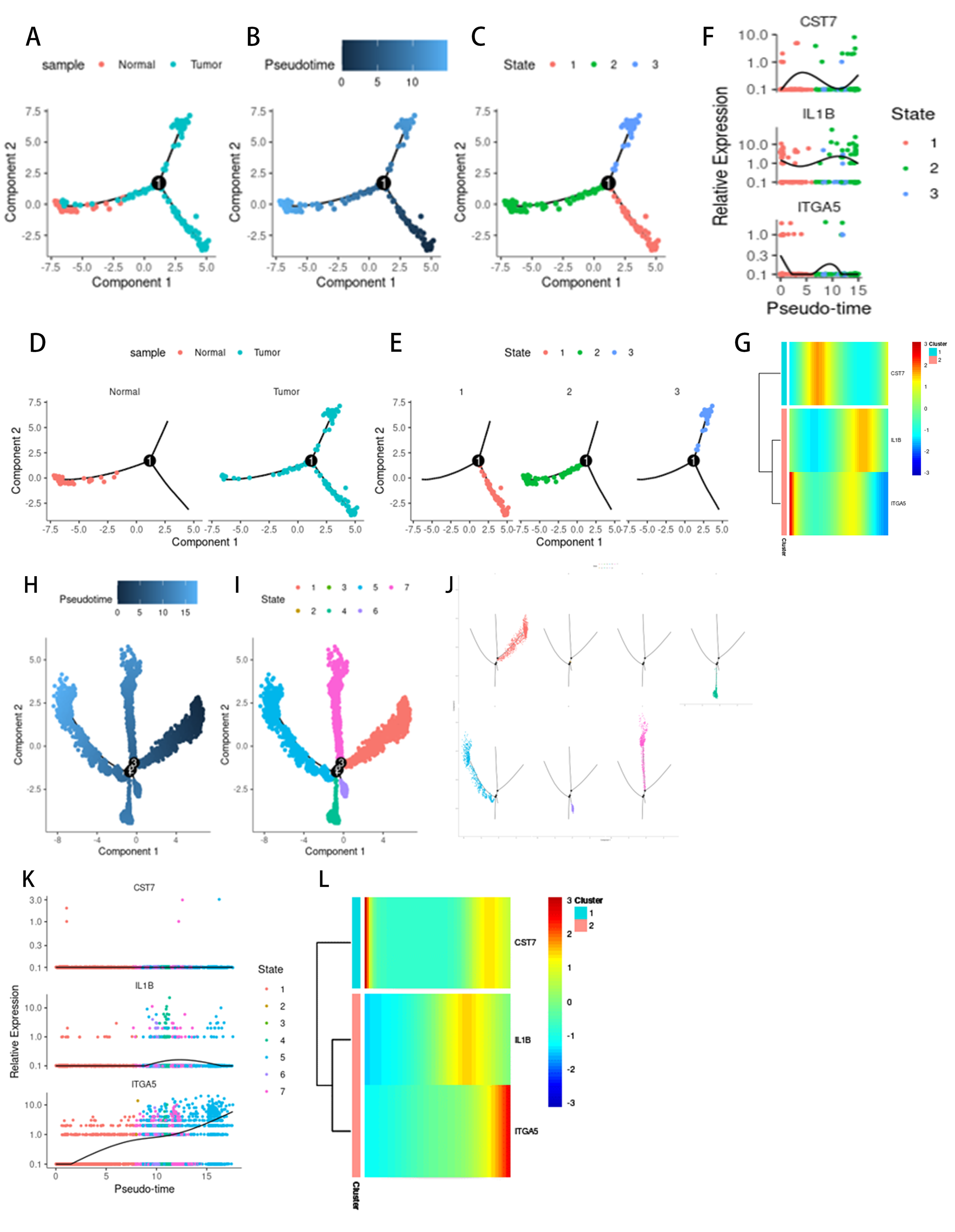

Supplement: Supplementary Figure 7 — Differentiation trajectories of macrophages and epithelial cells. (A) The distribution of normal and tumor cells along the macrophage differentiation trail. One differentiation node is generated. (B) The time distribution on the differentiation trajectory of macrophages. The darker the color, the earlier the differentiation time. (C) Branches generated by macrophage differentiation locus, including 3 branches. (D) The distribution of tumor and normal cells on three branches. (E) Corresponding differentiation positions of cells on the three branches. (F) Expression levels of IL1B, CST7 and ITGA5 in three branch cells. (G) Heat maps of expression patterns of IL1B, CST7 and ITGA5 on macrophage differentiation trajectories. (H) The time distribution of epithelial cell differentiation tracks, the darker the color, the earlier the differentiation. (I) The branching of epithelial cells along the differentiation path, with a total of 7 branches. (J) The corresponding position of cells on each branch of the epithelial differentiation pathway. (K) Expression levels of IL1B, CST7 and ITGA5 in 7 branch cells. (L) Heat map of expression patterns of IL1B, CST7 and ITGA5 in epithelial cell differentiation tracks. [file Image_7.tif]
